# Brain Imaging in Gambling Disorder

**DOI:** 10.1007/s40429-015-0063-x

**Published:** 2015-07-12

**Authors:** Saskia Quester, Nina Romanczuk-Seiferth

**Affiliations:** Department of Psychiatry and Psychotherapy, Charité-Universitätsmedizin Berlin, Charité Campus Mitte, Charitéplatz 1, 10117 Berlin, Germany

**Keywords:** Pathological gambling, Reward, Loss avoidance, Loss aversion, Brain volume, Functional connectivity, fMRI

## Abstract

Gambling disorder recently was reclassified under the category “substance-related and addictive disorders.” With regard to the diagnostic criteria, it overlaps a great deal with substance use disorder, i.e., loss of control, craving/withdrawal, and neglect of other areas of life. However, the gambling disorder symptom “chasing one’s losses” is the only criterion absent from substance use disorder. Therefore, special forms of reward (i.e., gain/loss) processing, such as the processing of loss avoidance and loss aversion, have just recently attracted attention among gambling disorder researchers. Because gambling disorder might be considered an addiction in its “pure” form, i.e., without the influence of a drug of abuse, investigating brain volume changes in people with this behavioral addiction is an important task for neuroimaging researchers in exploring the neural signatures of addiction. Because the brain is a complex network, investigation of alterations in functional connectivity has gained interest among gambling disorder researchers in order to get a more complete picture of functional brain changes in people with gambling disorder. However, only a few studies on brain structure and functional connectivity in gambling disorder have been performed so far. This review focuses on brain imaging studies of reward and loss processing, with an emphasis on loss avoidance and aversion as well as brain volume and functional connectivity in gambling disorder.

## Introduction

Gambling disorder is a psychiatric disorder characterized by maladaptive and excessive gambling behavior. Because this disorder shares clinical characteristics, as well as common cognitive and personality features, neurobiologic processes, and genetic vulnerability, with substance use disorders [1–5], it has been considered a behavioral addiction [2]. In the fifth edition of the *Diagnostic and Statistical Manual of Mental Disorders* (DSM-5) [6], gambling disorder is classified under “substance-related and addictive disorders.”

Theories on substance-related addiction (reward deficiency hypotheses [7], incentive-sensitization theory [8, 9], impaired response inhibition and salience attribution (I-RISA) [10]) stress the role of the prefrontal cortex and the mesolimbic reward system (especially the ventral striatum) in the development and maintenance of addictive behavior. Accordingly, neuroimaging research on substance use disorder has demonstrated changes in frontostriatal circuits at the brain’s structural and functional levels [11–13]. Regarding gambling disorder, neuroimaging studies are not as numerous as those for substance use disorders, although they also have reported changes in reward processing and prefrontal function (for reviews, see, e.g., [14, 15]).

Because substance use disorders have repeatedly been associated with altered dopamine transmission within the striatum in positron-emission tomography (PET) studies [16, 17], this neurotransmitter also has become the main focus in the investigation of neurochemical alterations in gambling disorder. Indeed, PET studies in gambling disorder have failed to detect significant alterations in dopamine transmission in the striatum as compared with controls [18–23] but found correlations with impulsivity, gambling severity, and monetary loss within the gambling disorder groups [18, 19, 21, 22]. Parkinson’s disease with gambling disorder behavior has been associated with increased dopamine release in the ventral striatum when compared with Parkinson patients without gambling behavior [24].

The diagnostic criteria for gambling disorder overlap largely with those for the substance use disorders [6]. Main symptom clusters (i.e., loss of control, craving/withdrawal, and neglect of other areas of life) may be linked to common experimental paradigms applied in neuroscientific research [25]. Regarding the symptom cluster “loss of control” (i.e., unsuccessful efforts to control, cut back, or stop gambling), studies at the behavioral level report diminished executive functioning with diminished response inhibition and cognitive flexibility [26, 27], as well as impaired decision making in tasks such as the Iowa gambling task [1, 28–30], in gambling disorder patients and problem gamblers. In line with these observations, functional magnetic resonance imaging (fMRI) studies found altered task-related brain activity in the prefrontal regions of gambling disorder patients and problem gamblers during a Stroop task [31, 32], during response inhibition [31, 33, 34], in the Iowa gambling task [35], and during an alternation learning task [36]. Neuroimaging studies on decision making also used probability and delay discounting and reported activity changes in the prefrontal and parietal cortex as well in reward areas (e.g., ventral striatum and caudate) [37–39].

The symptom cluster “withdrawal and craving” (e.g., restless/irritable when attempting to cut down or stop gambling and gambles when feeling distress) may be related to cue reactivity studies, because withdrawal and craving may be induced by addiction-related cues in substance use disorder because of sensitization of the mesocorticolimbic system [9]. Cue reactivity in gambling disorder has been associated with increased arousal and an increased craving for gambling [40, 41]. So far, only a few studies have investigated brain responses to gambling-related cues in gambling disorder patients and problem gamblers. Whereas one study observed a diminished urge-related brain response in the orbitofrontal cortex, basal ganglia, and thalamus [42], other studies found increased activity in the prefrontal and limbic structures, including the dorsolateral prefrontal cortex, anterior cingulate, ventral striatum, and parahippocampal gyrus [34, 43, 44]. Miedl et al. [45] found an influence of gambling-related cues, which modulated striatal value coding, on delay discounting.

The symptom cluster “neglect other areas in life” (e.g., is often preoccupied with gambling) may be related to alterations within the brain’s reward system with an overestimation of the short-term value of gambling-related reward and an underestimation of long-term losses [25]. Reward processing in this sense includes both the processing of gains and losses, especially in the context of gambling, in which losses are a potential consequence and can be avoided by winning or rejecting a gamble. Accordingly, fMRI studies observed activation changes in response to monetary reward and loss in ventral or medial prefrontal areas and the ventral striatum [46–49]. These findings, however, are not consistent: Miedl et al. [50], for example, did not find differential activation of the ventral striatum during monetary reward in problem gamblers or controls. The gambling disorder symptom “chasing one’s losses,” the only criterion of gambling disorder not present in substance use disorder, is related to an altered reward (i.e., gain/loss) processing. A special form of reward processing is the avoidance of loss since it might be considered as a negative reinforcement. Due to a successful avoidance of a negative consequence, subjectively experienced tension or stress caused by a potential loss is released. Moreover, changes in the sensitivity to losses (loss aversion) might also be a relevant aspect of loss processing for gambling disorder since gambles are always associated with a potential gain and a potential loss of money, which have to be weighed up against each other. This review focuses on brain imaging studies on gain and loss processing in gambling disorder, with an emphasis on loss avoidance and aversion.

Besides the investigation of task-related brain activation, other imaging modalities measuring important neurobiologic correlates, such as brain structure and functional connectivity, especially during rest, have gained interest in gambling disorder research. Brain structure changes have been observed widely in substance-related addictions [51–54]. In particular, alcohol dependence is accompanied by extensive volume loss [11], which most likely is a result of the direct toxic effect of ethanol on the brain [55]. Because gambling disorder may be considered an addiction in its “pure” form, i.e., without the influence of a drug of abuse, investigation of addiction-related brain structure changes and therefore neural signatures of addiction is possible.

With respect to functional connectivity, resting-state fMRI studies on substance use disorder have reported altered patterns of connectivity between cognitive control nodes, such as the lateral prefrontal cortex, anterior cingulate cortex, and parietal areas [56–58], and alterations in connectivity from the ventral striatum [57, 59–62]. With regard to gambling disorder, only a few studies on brain structure and functional connectivity have been performed so far, which is another key aspect of this review.

## Gain Processing

One task that is often used in fMRI to investigate monetary reward is the monetary incentive delay task [63]. The task allows the measurement of brain responses while the participant is asked to respond rapidly to a target in order to gain money or to avoid money loss. Applying this task to gambling disorder, Balodis et al. [46] reported reduced ventral striatal activation during gain anticipation as well as reduced ventromedial prefrontal activity during gain outcome. In line with these findings, Choi et al. [64] observed reduced activation of the ventromedial caudate nucleus during anticipation of gain in gambling disorder patients. These results underline the assumption of reduced reward sensitivity in gambling disorder. Accordingly, a study comparing a monetary reinforcer and a reinforcer with erotic content between gambling disorder patients and controls during reward anticipation and outcome [65] reported diminished brain activity in the ventral striatum of gambling disorder patients to cues predicting erotic stimuli as compared with controls. In a comparison of monetary and erotic cues (anticipation phase) within the gambling disorder group, these participants also showed a blunted brain activity to erotic stimuli, suggesting an imbalance with differential sensitivity to monetary versus nonmonetary reward in gambling disorder patients during reward anticipation. During reward outcome, gambling disorder patients showed increased activation in the posterior orbitofrontal cortex to monetary gain outcome compared with controls. In a modified version of the monetary incentive delay task (points instead of money to express the amount of reward) [66•], gambling disorder patients did not differ from controls in striatal activation during reward anticipation but displayed reduced activation in the insula, which was negatively related to the duration of the illness, suggesting that this alteration may result from excessive gambling.

Fauth-Bühler et al. [67••] investigated effort-dependent monetary reward processing in gambling disorder and found no difference during reward anticipation compared with controls. However, patients with higher scores on the Beck Depression Inventory showed increased insula and dorsal striatum activity during monetary gain outcome compared with patients with lower scores, suggesting that gambling disorder patients with depressive symptoms have more pronounced alterations in reward processing. Furthermore, this result underlines the importance of considering comorbid depressive symptoms in patients with gambling disorder.

Van Holst et al. [49] reported increased activity in the reward system while using a guessing task with monetary reward anticipation and value coding: problem gamblers showed stronger activation in the bilateral ventral striatum and left orbitofrontal cortex, associated with gain-related expected value. Accordingly, in the study by Worhunsky et al. [68•], gambling disorder patients performed a simulated “three-wheel” slot machine task and showed increased activity in the ventral striatum during reward anticipation.

At first glance, these studies seem to report discrepant findings regarding increased or decreased activation patterns in reward-related brain areas. However, in tasks using actual gambling cues [49, 68•], reward-related areas were more engaged, whereas in tasks applying more abstract monetary (e.g., numbers and points) or nonmonetary rewards (erotic stimuli and stimuli of high personal relevance), diminished reward-related activation or no activation changes during anticipation were observed [46, 64, 65, 66•, 67••, 69]. These findings are in line with those of studies on substance use disorders demonstrating enhanced brain activation in response to drug-associated stimuli [70, 71] and diminished brain activation in response to conventional reinforcers [72, 73].

## Loss Processing

Gambling disorder patients show impairment in the processing of loss information in risky situations [28] and of aversive stimuli [74], underscoring the importance of this aspect of reward processing in gambling disorder. Using a probabilistic learning task, di Ruiter et al. [47] observed reduced brain activity in the ventromedial prefrontal cortex during monetary losses in gambling disorder patients compared with controls. Balodis et al. [46] and Choi et al. [64] investigated loss anticipation, as well as loss outcome, using the monetary incentive delay task. Balodis et al. [46] found reduced ventral striatal activation during loss anticipation, as well as reduced insula activation during loss outcome, in gambling disorder patients. Choi et al. [64] observed reduced activation of the ventromedial caudate nucleus during anticipation of loss in gambling disorder patients. The aforementioned slot machine task used by Worhunsky et al. [68•] also allowed the investigation of a special form of loss processing—the processing of near-miss outcomes (unsuccessful outcomes that are almost a win and recruit reward-associated areas) [75, 76]: gambling disorder patients demonstrated diminished ventral striatal activation during near-miss outcomes compared with controls. Moreover, gambling disorder patients showed a generally blunted brain activity in response to losses, suggesting that near-miss and loss outcomes may be less salient in gambling disorder. The authors concluded that repeated exposure to near-miss and loss outcomes may influence/blunt brain responses over time [68•].

Studies performed to date have focused mainly on the processing of loss outcomes [46, 47, 64], whereas brain activation during successful loss avoidance has been neglected. This aspect of loss processing may be especially relevant for understanding of maladaptive behavior in gambling disorder, because it may serve as a negative reinforcement that in turn implies increased extinction resistance (i.e., increased persistence of operant behavior) compared with positive reinforcement [77]. In a recent study, we used a monetary incentive delay task and directly compared gambling disorder with alcohol-dependent patients [78••]. The gambling disorder patients demonstrated greater activation of the ventral striatum during loss anticipation compared with controls and alcohol-dependent patients, whereas activation in the right ventral striatum and right medial prefrontal cortex was diminished during successful loss avoidance compared with controls. Our findings suggest that loss-indicating stimuli are processed differently in gambling disorder and alcohol-dependent patients. Moreover, altered salience attribution to impending losses as well as loss avoidance may contribute to symptoms such as “chasing” behavior in gambling disorder patients. Loss chasing may be an important factor in the development of gambling disorder, as gamblers often meet this criterion in the absence of any other gambling disorder criteria [79], and already has been observed in neuropsychological testing in gambling disorder patients [80].

Another important aspect of loss processing is loss aversion: the phenomenon that a unit of possible loss subjectively weighs more than a unit of possible gain [81], i.e., a tendency to be more sensitive to losses than to gains. At the neural level, loss aversion has been associated with brain activity in the ventral striatum and medial prefrontal cortex [82, 83]. Decreased loss aversion might account for loss-chasing behavior in gambling disorder; our group has examined the neuronal correlates of this phenomenon in this disorder. In an fMRI loss aversion task similar to that used by Tom et al. [83], gambling disorder patients displayed reduced loss aversion on the behavioral level, as well as stronger increasing activation with growing losses in superior parietal lobule in patients (alcohol dependent and gambling disorder patients grouped together) compared with controls (Genauck et al., unpublished data).

## Brain Volume

To date, only a few brain imaging studies on brain volume in gambling disorder have been published. Two voxel-based morphometry (VBM) studies [54, 84] found no gray matter alterations on a whole-brain level. As for [54], subtle alterations might have been overlooked because a stringent correction for multiple comparisons across a whole brain image might be too conservative, especially in case of a priori hypotheses. Two diffusion tensor imaging studies reported white matter microstructural abnormalities in gambling disorder patients [84, 85], suggesting alterations in structural brain connectivity. Another approach is the use of regions of interest due to a priori hypotheses. Rahman et al. [86••] focused on the bilateral hippocampus and amygdala, because the behavioral inhibition system (BIS) has been associated with volume alterations in these brain structures [87]. The authors found reduced volume in the left hippocampus and right amygdala of gambling disorder patients compared with controls and a positive correlation between BIS scores and left hippocampal and left amygdalar volumes in the gambling disorder group. In a recent VBM study [88••], we used the prefrontal cortex and ventral striatum as regions of interest because of the importance of these brain regions in gambling disorder and their association with reward processing and self-control/inhibitory control [63, 89, 90] and observed higher volume in the right prefrontal cortex and right ventral striatum in gambling disorder patients compared with controls. Accordingly, with respect to a relationship to excessive behavior, increased volume was found in the left ventral striatum in adolescents who are frequent video game players [91] and in the right caudate and right nucleus accumbens in people with internet gaming disorder [92]. In a shape analysis of the data from our VBM study [88••], gambling disorder patients showed hypertrophy of the bilateral putamen and a positive correlation between gambling severity and hypertrophic shape alterations in this brain area, highlighting the association of subcortical brain volume alterations with the pathologic state [93]. However, whether the brain volume alterations reported by the different studies are a predisposition for gambling disorder or a sign of neuroadaptive changes due to the excessive gambling behavior still must be clarified. Nevertheless, training studies have shown that extensive experience with a certain behavior may enlarge associated brain structures [94–98]. Therefore, brain volume alterations in gambling disorder patients may represent a neuroadaptive response to excessive gambling and reinforcement processes in a gambling context. Thus, studies focusing on brain structural and functional alterations related to learning processes in a specific conditioning context (e.g., gambling environment) are a promising approach for future research in this field.

## Functional Connectivity

Because the brain is considered a complex network [99, 100], focusing on brain activation within specific brain regions is not sufficient to understand the neurobiological mechanism of disorders such as gambling disorder. To accommodate the idea of a network, neuroimaging studies started to investigate functional connectivity during certain tasks or during the resting state in gambling disorder. Based upon a priori hypotheses, brain activity within regions of interest (i.e., seeds) is correlated with brain activation in all other voxels of the brain to determine the strength of functional connectivity between brain regions. However, studies investigating functional connectivity between brain areas in gambling disorder are rare. Recently, two studies observed changes in functional connectivity of different striatal areas during inhibition and decision making in participants with gambling disorder [101, 102]. Van Holst et al. [34] investigated response inhibition in problem gamblers during an affective go/no-go task and further analyzed the data regarding functional connectivity with seed regions in the inferior frontal cortex and caudate [102]. Problem gamblers showed less functional connectivity between the left caudate and occipital cortex during response inhibition in the neutral condition (go and no-go signals were neutral pictures) compared with controls, which was interpreted as the controls having more visual attention and therefore better performance than the gamblers. In contrast, during response inhibition in the positive condition (go signals were positive pictures and no-go signals were neutral pictures), problem gamblers showed stronger functional connectivity between both brain regions while making fewer response inhibition errors than controls. During response inhibition in the negative condition (go signals were negative pictures and no-go signals were neutral pictures), stronger functional connectivity between the left caudate and the right anterior cingulate cortex was found in the problem gamblers compared with controls. As proposed by the authors, increased functional connectivity between a ventral affective system and a dorsal executive system in problem gamblers during response inhibition suggests facilitation of the dorsal executive system when affective (positive or neutral) stimuli are present. Peters et al. [101] reanalyzed the data of Miedl et al. [37] to examine the functional connectivity in gambling disorder patients during value-based decision making (delay discounting and probability discounting) by using ventral striatal seeds. The gambling disorder patients showed stronger functional connectivity between the ventral striatum and amygdala across delay and probability discounting trials. The authors interpreted this increased connectivity within limbic circuits as a possible contribution to impaired impulse control.

Another task-related functional connectivity study was conducted by van Holst et al. [103]. In this study, changes in functional connectivity in regular gamblers and nongamblers were investigated while the participants played a slot-machine game, with winning and near-miss outcomes and the interaction of near-misses by personal choice. By using striatal seed regions, the investigators observed an overall increase in connectivity to the left orbitofrontal cortex and posterior insula and a negative correlation between gambling severity and connectivity to the left anterior cingulate cortex for a liberal statistical threshold during winning. For near-miss events (when interacting with personal choice), connectivity to the insula correlated positively with gambling severity. This study demonstrated functional connectivity changes from reward-related areas during winning and near-miss events. More severe gambling problems were associated with lower functional connectivity within reward areas.

Furthermore, Jung et al. [104••] investigated default mode network activity in gambling disorder patients. Default mode activity, intrinsic neural activity during the resting state, correlated positively with brain activity in the posterior cingulate cortex [105, 106]. With a seed in the posterior cingulate cortex, gambling disorder patients displayed less default mode connectivity to the left superior frontal gyrus, right middle temporal gyrus, and precuneus than healthy controls. Further, decreased functional connectivity between the posterior cingulate seed region and the precuneus was correlated with the severity of gambling disorder symptoms. Because default mode network alterations also have been observed in substance use disorders [57, 107], the authors concluded that decreased functional connectivity within the default network may be a shared neurobiologic mechanism in gambling and other addictive disorders. Moreover, this study also demonstrated that in a task-absent condition, gambling disorder is associated with an alteration in the functional organization of the brain.

In a resting-state functional connectivity study [108••], we investigated connectivity changes from a seed in the right anterior prefrontal cortex and right ventral striatum based on the results of our VBM study [88••]. Gambling disorder patients demonstrated increased functional connectivity from the right anterior prefrontal cortex to the right striatum and decreased connectivity to other prefrontal areas compared with controls. Accordingly, the right ventral striatum demonstrated increased connectivity to the right superior and middle frontal gyrus in the gambling disorder patients compared with controls. Neuroimaging studies have shown that extensive experience with a certain behavior may alter brain activation [98, 109]. Excessive gambling behavior therefore may result in neuroadaptive processes, such as increased functional connectivity. In turn, increased functional connectivity might reflect facilitated bottom-up information processing (i.e., from ventral striatum to prefrontal cortex) by training effects or a facilitated transmission of action impulses from the prefrontal cortex to the ventral striatum in case the prefrontal cortex also is involved in the planning and motivating of gambling behavior. Because we found decreased connectivity between different prefrontal regions, it is unlikely that increased functional connectivity between the prefrontal cortex and ventral striatum reflected an enhancement of self-regulatory competencies over gambling impulses. Increased connectivity between the prefrontal cortex and ventral striatum [57, 62] and decreased prefrontal interhemispheric connectivity [56] also have been observed in substance use disorders, underlining the close relationship to substance-related addictions.

Because the aforementioned connectivity studies used seed regions due to certain a priori hypotheses, they were not able to examine all possible network organization alterations in gambling disorder. By using graph-based methods, which consider brain regions as nodes on a graph [110], brain network characteristics can be explored without any assumptions and without limiting the results to certain seeds. Tschernegg et al. [111••] applied a graph-theoretical approach to resting-state data from gambling disorder patients to examine network properties on the global and nodal levels. Gambling disorder patients demonstrated no alterations in global network properties. At the nodal level, however, they showed alterations in network properties in the paracingulate cortex and the supplementary motor area. At an uncorrected threshold level, alterations also were observed in the inferior frontal gyrus and caudate. Moreover, functional connectivity between brain regions was computed: gambling disorder patients demonstrated increased functional connectivity between the left caudate and right anterior cingulate. Functional connectivity also was increased between frontal regions and between frontal and temporal regions but decreased between the left amygdala and left subcallosal cortex in gambling disorder patients compared with controls. Similar to our study’s findings, those of Tschernegg et al. [111••] suggest brain network alterations in gambling disorder with respect to the reward system and areas related to executive functions.

## Conclusion

The main focus in neuroimaging research in gambling disorder is on reward processing because of its role in addictive behavior. Important aspects of reward and loss processing are loss avoidance processing and loss aversion, which might play an important role in loss-chasing behavior and therefore are an important research area in gambling disorder. Other neurobiologic correlates, such as brain volume and functional connectivity, are less examined in gambling disorder. Observations of brain volume seem to depend on a methodologic approach: VBM whole-brain analyses found no alterations, whereas region-of-interest analyses found volumetric changes in subcortical brain areas. Since gambling disorder is conceptualized as an addiction without the neurotoxic effect of a drug of abuse, these alterations might be related to addiction-related processes. Results of functional connectivity studies are in accordance with task-related findings in decision making and reward processing, because these studies found changes in reward- and control-related brain networks.

Neuroimaging research in gambling disorder has been limited because often only male gambling disorder patients or problem gamblers have been studied; therefore, the results are not transferable to the general gambling disorder population. Male and female gambling disorder patients differ with regard to symptom pattern, sociodemographic, and clinical parameters [112], so gender differences also may exist in neurobiologic correlates. Moreover, it remains unclear which alterations are a predisposition of gambling disorder and which develop with maladaptive gambling behavior. Therefore, future research also should apply longitudinal designs.
